# Diamond Open Access in the quest for interdisciplinarity and excellence

**DOI:** 10.3325/cmj.2017.58.261

**Published:** 2017-08

**Authors:** Srećko Gajović

**Affiliations:** University of Zagreb School of Medicine, Croatian Institute for Brain Research, Zagreb, Croatia *srecko.gajovic@hiim.hr*

Being a small independent scholarly journal owned by 3 Croatian universities, the *Croatian Medical Journal (CMJ)* needs to compete with many other biomedical journals, mostly belonging to the big commercial publishers. With the 2016 impact factor of 1.619, the *CMJ* seems to fare very well in this competition, positioning itself in the second quartile of its group. The *CMJ* is currently 25 years old and has a solid reputation among biomedical researchers – one can speak about a success story in what was achieved.

Several characteristics of the *CMJ* contribute to this success story. Among them, I would like to highlight the fair relationship to the biomedical research community, reflected in its Diamond Open Access. Being Diamond means that the journal does not earn money from the business, which is an immediate distinction from the commercial publishers (1). We at the *CMJ* do not charge readers, nor do we charge authors. The articles published in the *CMJ* are freely accessible at the journal’s web page, www.cmj.hr, and at one of the major repositories of biomedical articles, PubMed Central. If an interested reader performs a PubMed search, the full-text of an article published in the *CMJ* is only a click away. This allows for an unhindered visibility of the published articles. Regrettably, similarly to the commercial publishers, we do not offer any rewards to our reviewers, which places a serious constraint on manuscript processing. Finding experts willing to do this demanding work becomes more and more difficult (2). In my opinion, the commercial publishers should as soon as possible start to share their income with the reviewers doing the job of major importance for them. If a manuscript processing fee is charged to the authors (in case of the Gold Open Access) and the crucial step in manuscript processing is a reviewer opinion, then charging a processing fee for something completely different, without taking the peer-review process into account, is not fair to any of the participants in the science publishing process (apart from the publishers themselves). Subsequently, if no reward is provided to reviewers, the only fair approach is a Diamond Open Access, where in the same way as the reviewers are providing their opinion for free, the *CMJ* provides their services to the authors for free and allows the readers to read the articles for free. Obviously, there are some costs to sustain the journal’s operations. In the *CMJ*’s case, these costs are covered by the grant from the Croatian Ministry of Science and Education and by public universities, the owners of the *CMJ*. As grants are the major source of funding for authors to cover the article processing fees (ie, for journals belonging to Gold Open Access, where authors pay and readers read articles for free), the sustainability of our journal actually relies on the same financial resources (agency grants) being payed directly to the journal. If a grant is given directly to the journal, it makes it significantly more efficient than if it was distributed as an individual article processing fee. Moreover, it offers a way to avoid the substantial profit margins present in the commercial publishing industry at the expense of the research community. Subsequently, we argue that the commercial publishers need to share their income with their reviewers (if they want to charge and earn money during publishing process). Otherwise, if the above is not the case, the only fair approach is a Diamond Open Access, to which the *CMJ* adheres.

Another advantage of the *CMJ* is the vast scope of biomedical research it covers. At the first glance, this may be viewed as a disadvantage because we do not have a dedicated research community in the specialized filed, which would recognize the journal as an expert meeting point for emerging issues. Today’s digital environment and enormous advances in almost every medical field, particularly in pre-clinical research, regrettably make the concept of the safe harbor for the subspecialized experts where they would discuss their scientific interests not valid any more. The preclinical and clinical medicine nowadays intermingle, new approaches in one field influence another, and public health interventions need to be based on the wide understanding of medicine and society (3). The strength of the *CMJ* is that it offers room for inter-field and interdisciplinary communication, allowing for the articles cutting across different medical fields to be evaluated by reviewers of different expertise and to be visible to the readers of the different professional backgrounds. This has been exemplified in the Knowledge Landscapes column, where the question of empowering patients to find innovative medical knowledge has connected those working not only in the field of medicine, but also in the fields of philosophy, social science, law, and economy (4). The search for excellence should rely on two equally important approaches – on being meticulous and finding expert details characteristic of a particular field of research and on seeing the big picture and providing thoughts about general direction of the biomedical research development.

The future of the *CMJ* relies on its firm standpoint on research integrity, as emphasized in the recent Sarajevo Declaration (5), and its tradition to scrutinize together with the reviewers every detail of submitted manuscripts. This effort is absolutely necessary in order to publish only excellent research expected to make impact in the field. On top of this, the *CMJ* uses innovative strategies to contribute to the advancement of the scholarly publishing and biomedical research. These currently include the Diamond Open Access, as an example of a fair operational model for a scholarly journal, and interdisciplinarity, as the major challenge in the biomedical research.
